# Exploring channels and gaps in information dissemination and acquisition among energy scientists and the public: The southeast Asian context

**DOI:** 10.1371/journal.pone.0273626

**Published:** 2022-08-29

**Authors:** Langcheng Zhang, Shruti Malviya, Edson C. Tandoc, Shirley S. Ho

**Affiliations:** Wee Kim Wee School of Communication and Information, Nanyang Technological University, Singapore, Singapore; Shenzhen University, CHINA

## Abstract

This study investigates the flow of energy-related information, which plays a vital role in promoting the public understanding and support for various energy sources. Through 12 focus group discussions with the public and energy experts, this study found that energy information flows from scientists to the public through both direct (e.g., roadshows, scientists’ blogs) and indirect (via agents, e.g., school, news media) channels. However, communication gaps remain between scientists and the public. First, the public commonly obtains information from personal experience and the media but not directly from scientists. Second, while the public stressed the importance of mass media and social media, only a few experts reported writing news commentaries or making social media posts about energy. Third, while scientists emphasize their relationships with the government and other agencies in disseminating information, the public shows relatively weak trust in these agencies. Implications are made for future research and public communication on energy issues.

## Introduction

The COVID-19 pandemic saw reductions in the energy consumption across major cities in Asia and across the world. In particular, research found that restrained use of traditional energy sources during the pandemic significantly reduced air pollution [1–4]. But as the world enters a post-COVID-19 industrial world, where energy consumption surges back to pre-pandemic rates, the need for clean and sustainable energy sources becomes more salient. This occurs against the backdrop of changing political dynamics, such as Russia’s invasion of Ukraine, that has hampered oil supply and has brought fluctuations to the global oil market [5, 6]. These highlight the necessity of adopting alternative resources to overcome environmental, economic, and political risks caused by a reliance on traditional energy.

Therefore, intergovernmental organizations and nature conservationists have been pushing for new energy resources, not only as a way to tackle environmental problems, but also to encourage economic growth through energy efficiency and sustainability [7]. However, acceptance of new energy has not been easy in many countries partly due to weak support from the public [8–10]. A substantial barrier was found in the information asymmetry of energy usage, alternatives, and potential ways of upgrading efficiency [11]. To counter this asymmetry, a vital step is to ensure that the public receives accurate energy-related information.

Science communication studies have examined the public’s role through two dominant public engagement models; one assumes the public to be deficient in knowledge and thus needs to be educated, while the other sees the public as having the potential to be actively involved [12]. While the applicability of these models largely depends on various political and cultural contexts [13], most studies have focused on the Western context [14], leaving a missed opportunity in understanding science communication in a global environment.

This study seeks to explore how energy-related information flows in a non-western context, focusing on Southeast Asia. This brings into focus two objectives. First, with heavy reliance on fossil resources, Southeast Asian governments are promoting renewable energy as the region is endowed with a rich variety of natural resources [15–17]. However, social pressures hinder the implementation of new energy policy in these countries; for instance, local communities are often uncooperative [18, 19]. Second, attitudes toward energy sources are shaped by the range of information the public gets. Thus, it is important to understand how energy experts disseminate information about energy sources as well as how the public obtains information about energy sources.

Focusing on the three neighboring countries, Singapore, Malaysia, and Indonesia—whose energy supply mix are interconnected; Singapore, for example, gets some of its energy supply from these two countries—this current study draws from a series of focus group discussions to understand 1) how scientists disseminate energy information, 2) how the public acquires energy information, and 3) how the approaches used by scientists to disseminate information compare with those used by the public to acquire it. The last question aims to reveal the information gaps between scientists and the public. Only by detecting the gaps can we better inform future efforts to focus on approaches that may be under-utilized.

### Literature review: Scientists’ approaches to communicating science

Communication scholars have often delved into the relationship between scientists and the public in two dimensions—examining the direction and stages involved in the communication process. Regarding the direction, studies have suggested two models—a one-way or top-down model and a two-way dialogical model, each involving varied levels of public engagement [12]. The one-way, or knowledge deficit model, presumes that the public’s scepticism towards science is due to inadequate information, so scientists need to provide information to an otherwise knowledge-deficient public in a one-way approach. While this model focuses on building awareness and knowledge among the public, it does not lead to increased participation [20].

As a solution, scholars proposed dialogue and participatory models, emphasizing public engagement in science beyond mere communication to consultation and participation [21]. In the two-way processes, the priority of communication shifts from educating the “scientifically illiterate public” to the need for the public to participate, establishing the public as competent to understand and interact with the scientists [22]. Despite the popularity in recent years [12], gaps remained in two-way models such as negative comments on public platforms [23] and scientists’ disconnect from the public [24].

The scientist-public communication is a matter not merely of direction but also involves specific processes and stages. In fact, studies have shown that different models can coexist [12], making it crucial to investigate what stages are involved in each process and to what degree the process embraces authority or interactivity.

### Literature review: Stages of information flows

Scientific information flows either linearly through a specific medium or dispersedly through multiple channels [25]. Studies have divided the flows into one-step [26], two-step [27], multi-step [28], and mediated flows [29]. Nevertheless, these discussions were always of a normative nature. An empirical test becomes imperative to determine which flow depicts a more realistic relationship between scientists and the public.

**Direct flows** involve direct messaging from scientists to the public through channels more relevant and efficient to the scientists [26]. Scientists remain in direct contact with the public through talks and discussions [30] or blogs, or social media [31]. However, as a drawback, information in these direct channels may be too technical for laypersons to understand [32], hindering the effectiveness of direct communication. Therefore, scientists must develop skillful strategies to engage with the public directly [33].

**Indirect flows** involve indirect messaging from scientists to the public through external agents. These agents convey scientific information in simple terms to the public, thereby facilitating the understanding of an otherwise technical concept [29]. For instance, mass media can act as an agent in the two-step model [27], and websites, research papers, as well as social media in multi-step models [28]. These models also allow scientists to communicate to the public through other social sectors, including the government, interest groups, non-governmental organizations, journalism, and educators [21, 25, 34]. The key to understanding the indirect flows is information agents serving each process.

As information comes from scientists and is distributed through multiple channels, it is necessary to examine where it goes and how different information agents are located on the extended map of scientific information. Public communication of scientists has been widely investigated on issues of environment and climate change [34, 35]. However, energy communication is still an emerging field with much uncertainty to clarify [36]. Therefore, this paper seeks to answer:

**RQ1:** How do scientists disseminate energy information to the public?

### Public’s information sources about energy

Various studies have investigated how different social actors contribute to the accessibility of science to the public. These actors include schools [37], media [38], industries [39], tourism [40], policy consultants and audits [41, 42], and grassroots activists [43]. Drawing from the notion of the agents of socialization, this paper will identify and categorize these agents in the case of energy-related information and evaluate their efficiency and reliability with empirical evidence.

### Agents of socialization and information acquisition

Socialization refers to the process where individuals are taught knowledge, values, skills, and behavioral patterns needed for competent functioning in the community and culture that one grows up with [44]. Information acquired passively [45] or actively [46] plays a prominent role in socialization—it is processed in specific cognitive modules and evolves to accomplish essential tasks in future socializing [47]. A critical function of socialization agents is to provide information to individuals. In line with the social learning theory, socialization agents include, but are not limited to, family [48], school [49], community [50], peers [51], workplace [52], media [53], and the Internet [54].

Some studies have demonstrated the role of socialization agents in disseminating scientific information. The first group of agents exerts influence at an **interpersonal level**. Studies have documented the role of primary schools [55], high schools [56], and universities [57] in offering children and young adults scientific courses. Through education, most people generate their basic knowledge about science and the ways they use it in the future [58]. The same goals can also be achieved by parental education [59] and daily activities [60] in the family setting. Everyday life [61], games [62], on-ground projects [63], and interpersonal communication [64] have also been seen to circulate information and boost the understanding of science among different people.

The second group of agents is the **mass media**. Traditionally, science seekers have relied on newspapers [65], broadcasts [66], and TV news [67]. Today’s news consumption habits involve the Internet [68], especially new media [YouTube, 69, Twitter, 70]. The public can receive scientific information as and when they interact with other actors. The online science literacy movement has witnessed an increasing rate of independent searches for scientific terms on Google [71] and Wikipedia [72]. In health information, social media have become a popular space for consumers to gather information about diseases and public health [73]. On all these accounts, it is reasonable to claim that agents assisting the public in acquiring information are comparable to those used for socialization.

Access to information helps in promoting environmentally friendly energy consumption habits, because accessibility can affect people’s knowledge which, in turn, may lead to pro-environmental behaviors [74]. However, the unequal distribution of information has put the public in an inferior position to other stakeholders [35]. To examine what sources are available to laypeople and how they navigate these sources among the information agents, we asked the following question:

**RQ2**: How does the public get energy-related information?

However, scientific information may not always accurately reach the public due to various reasons [75]. Two reasons stand out in the literature: scientists are not trained as routine public communicators [76], and the public has limited access to the scientists’ information, so they rely on third-party actors [77]. When there is a mismatch in information channels, the public will retain a low engagement with science [78], and the “not in my backyard” phenomenon may persist, where people show interest in scientific developments [79]. To identify information flow gaps, it is important to not only identify how energy experts communicate and how the public access energy-related information, but also to critically compare delivery and reception processes. Therefore, we also ask:

**RQ3**: How do approaches used by the public to acquire information compare with those used by scientists to disseminate it?

## Method

This study was based on online focus group discussions (FGDs) with energy scientists and the public separately, in Indonesia, Malaysia, and Singapore. FGDs can provide details related to complex topics such as information sources that require elaboration [80] through participant-led discussions and interpersonal interactions [81]. Besides, FGDs yield high ecological validity by stimulating natural, everyday conversation settings to ensue [82]. Like face-to-face FGDs, the online mode of FGDs is also effective in generating well-round insights of participants; additionally, it provides convenience for participants to join from anywhere regardless of geographical boundaries [83], especially during the period of data collection, marked by a global pandemic.

### Sampling and recruitment

Upon the ethics approval of Nanyang Technological University Institutional Review Board (NTU-IRB), we conducted three online FGDs with energy scientists and nine with the public, in Indonesia, Malaysia, and Singapore, between November 2020 and February 2021. A total of 104 participants were recruited using a mix of convenience, quota, and purposive sampling. Before each FGD session, participants provided their demographic information (see Table 1) in a pre-session questionnaire and signed an informed consent form.

**Table 1:** Demographics of focus groups.

FGDs	Group composition in each country		
	Singapore (n)	Malaysia (n)	Indonesia (n)
General public	10 (6 males, 4 females) Aged 21–39	8 (4 males, 4 females) Aged 21–36	8 (4 males, 4 females) Aged 20–38
	10 (5 males, 5 females) Aged 40–55	8 (4 males, 4 females) Aged 43–52	8 (4 males, 4 females) Aged 40–52
	10 (5 males, 5 females) Aged 56–74	8 (4 males, 4 females) Aged 56–73	8 (5 males, 3 females) Aged 59–70
Energy scientists	9 (7 males, 2 females) Aged ≥ 21	8 (5 males, 3 females) Aged ≥ 21	9 (8 males, 1 female) Aged ≥ 21

In recruiting the general public, participants were recruited door-to-door in a word-of-mouth manner. Participants were classified by generation according to Pew Research Centre’s [84] definition. Altogether 78 participants were recruited (n_Indonesia_ = 24; n_Malaysia_ = 24; n_Singapore_ = 30), all of whom were citizens/permanent residents. The minimum age threshold was determined by the legal voting age in each country. Considering Internet accessibility concerns, only residents in the capital cities were recruited (i.e., Jakarta in Indonesia, Kuala Lumpur in Malaysia, and Singapore). While public participants had varied educational and professional backgrounds, none held expertise in energy fields.

In recruiting energy scientists, participants were reached through telephone and email. To consolidate contacts with scientists, we created an initial sampling frame based on open information from organizations and their contacts. Additional participants were sought via snowball sampling. In all, 26 participants from Indonesia, Malaysia, and Singapore were recruited (n_Indonesia_ = 9; n_Malaysia_ = 8; n_Singapore_ = 9). They were local residents with two to 50 years of energy-related expertise in academia (Assistant Professors, equivalent or above), research institutes (senior scientists), or the energy industry (consultants, engineers, and managerial positions). Table 2 shows the specific areas of expert participants.

**Table 2:** Expert participants’ area of energy expertise.

Country	Areas of Expertise
Singapore	Nuclear power, solar energy, fossil fuels (natural gas, petroleum), bioenergy, wind energy, hydropower, geothermal energy
Malaysia	Nuclear power, solar energy, fossil fuels (natural gas, coal, petroleum), bioenergy, wind energy, hydropower, hydrogen energy
Indonesia	Nuclear power, solar energy, fossil fuels (natural gas, petroleum), bioenergy, hydropower, geothermal energy, ocean, and wave energy

### Procedure and moderation guide

Each session lasted approximately two hours online. Participants were compensated at the end of the session. The FGDs were conducted in the local languages. An experienced moderator moderated each session to encourage free-flowing discussions with the assistance of an assistant moderator who made notes and provided technical support. In the Singapore sessions, the moderators were two faculty members from the research team. In Indonesia and Malaysia sessions, we hired moderators with vast experience conducting FGDs in the respective countries and briefed them beforehand on the topics and standardized procedures.

During each session, the moderators followed the semi-structured topic guides prepared by the research team and translated into local languages by professional translators. Back-translation by native speakers was used to further improve accuracy. The guides involved a list of questions and prompts, such as asking what energy sources they are familiar with, how do they get information about these energy sources, as well as their perceptions of these energy and information sources, among others. Similar questions were asked across the two groups to allow comparisons, although unique questions were also included in each group (e.g., scientists were asked about their information dissemination strategies). For example, in the public sessions, participants were first asked to write down all the types of energy they knew; afterward, the key questions were asked to address where and how they learned about the energy sources they wrote. In the expert sessions, participants started by telling their experience in conveying energy-related information to the public and then continued by discussing what strategies they use to communicate energy issues. Other questions in the guides concerned participants’ trust and opinions about energy sources, which are not the focus of the present paper.

### Data analysis

The sessions were digitally recorded and transcribed verbatim. Team members checked English transcripts for discrepancies (e.g., typological errors). Non-English transcripts were translated into English by professional translators, with linguistic uncertainties clarified with the team (e.g., Bahasa Indonesia slang words). To ensure participants’ confidentiality, identifying information was concealed and substituted with alphanumeric codes in the transcripts.

Data analysis followed the constant comparative approach in grounded theory [85], an approach that has been broadly used in qualitative research [86]. In the open coding stage, two team members were trained and coded the transcripts separately, line-by-line, generating a list of codes about information sources. Each emerging code was compared to the preceding codes to determine if a new code is required, or if the next line suggests repeated codes, or if a previous code should be revised [87]. Then in the axial coding stage, the coders focused on examining the codes that emerged in the open coding stage, categorizing them into “conceptual bins” that describe themes emerging from the data [86, 88]. Through this two-stage coding process, the final categories of public information sources and scientists’ information outlets were generated. Finally, narratives were written for each theme with exemplars.

## Results

### Public communication among energy scientists

RQ1 asked about the different ways in which scientists disseminate energy information to the public. Scientists clarified the importance of conveying energy-related knowledge in growing public awareness, supporting energy management, and communicating potential risks. I4P6 stressed upon raising public awareness to suggest energy alternatives and consequences, “For me, the most important is the public’s awareness that conventional energy will finish quickly.”

Consistent with our analysis of the public, scientists also used the term “socialization” to describe their communication activities, as I4P8 said, “We often do socialization, sometimes with research and technology. We socialize them to various regions, to Bangka, and perhaps some provinces related with nuclear [sic].” Further, our analysis found that scientists’ socialization can be achieved through both direct contacts with the public and via information agents.

### Direct flow

One approach for scientists to directly reach the public was through on-ground projects. M4P3, based in Malaysia, recalled physically heading to villages and remote areas to give information to the local residents: “When I go to that particular village… We have to see what are current situations, and from there we can comment on what types of energy can be suitable particularly for them [sic].” M4P6 also spoke about promotional roadshows and tours in collaboration with the ministry: “Every time we are part of Ministry of Science’s road tour… Currently we don’t have nuclear energy in Malaysia, so we promote the technology [sic].”

Additionally, social media platforms are also adopted by some scientists to spread information, though this was not common in our interviews. I4P4, an expert from Indonesia, talked about using audio- and visual-based social media platforms:

“I have a podcast that focuses on the energy field. So, the main function of a podcast, Geoinsight, is to communicate things and information that might be basic to the public… It is the public communications on the media podcast and YouTube [sic].”

Interestingly, while a direct flow is always associated with interpersonal contacts in both physical and online settings, it is not the most sought-after route of energy information. Instead, scientists more often expressed the use of indirect flows, involving agents like mass media, education, the government, and other decision-makers to reach the public.

### Indirect flow

Here, scientists provide energy information to a third-party agent of socialization who, in turn, distributes secondhand information to the public. A frequently mentioned way was through professional teaching and peer socialization. Scientists share their findings among researchers, industry-based experts, and interested media professionals. By teaching college students and technicists, practical information is conveyed to industrial and private sectors. In these activities, however, it is not clear to the scientists whether the information ultimately reaches the masses. While they listed these activities as practices to disseminate information, they may not necessarily contribute to public information accessibility unless other agents further channel the information.

More efficient ways are through government, public sectors, and industries, like M4P1 described: “To all types of stakeholders in one time. To the industries that cover more than 1000 companies in Malaysia… Other times, communicate to the government on how and why we should annex this kind of law [sic].”

The government served as a key agent of scientific information. When scientists discussed disseminating their findings, they spoke more about sharing information with the government and policymakers rather than the public. S4P3 from Singapore said, “We regularly hold classes and courses for regional policymakers, where, obviously, I teach the part on solar, we’re trying to put it in perspective: I touch on nuclear, I touch on gas, and the other sources.” This shows the healthy and regular dialogues between researchers and decision-makers in Singapore, which is reaffirmed by S4P6 saying, “I’ve done forums, I’ve done conferences, I’ve done one-on-one talks with various, you know, decision-makers in the government.”

While not as evident or sought after, one more agent used in public communication has been mass media, including mainstream and social media. Stepping away from the more academic platforms such as scientific reports, journals, or forums, scientists have reported using mass media channels to disseminate information to the public. For example, scientists have increasingly used newspapers and news websites in their related fields. S4P8 added: “I’ve been writing policy briefs, commentaries, and also Channel News Asia interviews, and actually, I just finished one filming on Thursday with Channel News Asia.”

Another agent that comes to the forefront is the educational sector, whose association with experts has been traditionally strong, like I4P1 said, “I am from an educational campus, so I connect a lot with the educators. . . . We are a part of making the public concerned that there is an issue in the field of energy.” Participants illustrated their experiences of attending forums and discussions, writing papers, and making publications. Through these scholarly activities, scientific information reaches different levels of education as schools, colleges, and training sectors.

### Information sources of the public

RQ2 asked about the information sources that the public accessed for energy-related information. While some evidence suggested the direct flow of information through personal experience, an indirect flow of information through information agents remained consistent. In the interpersonal context, the information goes through human agents such as teachers, friends, and families. In contrast, in the mass media context, information is transmitted with the assistance of media agents, including books, news media, the Internet, etc.

### Personal experience

Daily activities and life experiences serve as a source of scientific knowledge in many fields [89]. In this study, participants reported knowing about energy through personal experiences. Most participants became familiar with traditional and new energy sources from direct experiences, such as seeing solar panels on the roadside (S2P8), Nuclear Agency in the Kajang city (M1P7), or windmills on the trip to Australia (S2P6).

Nevertheless, we found that these experiences are difficult for people to recall unless they were asked to do so. For example, when asked about information sources, S1P10 said, “It just seems like we know so well, like air that we breathe; I find it very hard to pinpoint exactly where.” As a result, such information is latent and needs a trigger for people to retrieve. For instance, I2P3 added wind to his list of energy when telling his life episode, “In 2019 I went to Dieng, to the Cikunir mountain. There is an electrical energy generator from geothermal…there was a windmill …oh, the wind can also be transformed into energy!”

Routine and accidental exposures to energy information have not only contributed to one’s knowledge but also led to active information-seeking through other channels. For instance, exposure to energy-related messages primed S3P10 with more interest in energy-related content in the media, as he said, “I think a lot of us watch Discovery, National Geo… I travel a lot as well, so I see alternate (energy) sources overseas, which kept me interested in the large windmills, the dams, etc. [sic]”

### Interpersonal settings

Unlike personal experiences which is often fragmented and random, interpersonal agents usually provide information systematically through social networks. Among the most mentioned agents, primary and secondary schools were especially pervasive in the discussion of young-age groups. In Singapore, some participants precisely mentioned school classes of Geography and Chemistry. S1P5 recalled, “For the combustion of fossil fuels or natural gas it’s through Chemistry; whereas the rest of the like uh so-called more environmentally friendly ones are like solar, wind, geothermal from Geography.”

Interpersonal settings also include universities and workplaces, where people get professional training or give professional services. Information from these agents is not available to the general population but only to those in energy-related fields. Here, educational agents range from polytechnics and universities in Singapore (S1P4) to online training courses and seminars provided in Malaysia (M2P8). The workplace agents are widely distributed across industries, including energy and engineering companies, NGOs, research entities, and even in therapist clinics, coconut businesses, or the stock market.

Knowledge about energy is also shared among family members, like I1P4 said, “Sometimes I tend to ask the husband or parents.” Schools provide scientific information not only to teenage students but also to their parents who get involved in the reciprocal process of family tutoring. This was particularly relevant to participants in the mid-aged groups, whose children took them along to explore new energy resources they had never encountered before. Sharing her experience, S2P7 said about her motivated information seeking:

“I still have a son in secondary school. So, when they learn geography and all, you get to learn all these terms too. So, as he asked me questions, I need to surf the net, so that’s how we find out more.”

Acquisition of energy information can either be active or passive. These examples show that active information seeking can be either task-oriented (tutoring) or interest-driven (TV or trips). Such motivated behaviors allowed participants to absorb information beyond the given context, guiding them to broaden their access to multiple media types.

Additionally, energy information was also acquired passively from information sharing among peer groups. Social network sites such as Facebook enabled users to share messages onto their homepages or to other users. Messaging apps have further facilitated information sharing through social networks. Like I3P4 said,

“From the (SNS) sharing we will know more complete information… There are those shared to my WhatsApp, for instance… For me, the main thing is indeed sharing, sent often by friends—my friends that have a lot of knowledge send it to me [sic].”

Lastly, scientific information is also shared by local community leaders. For example, I2P2 said, “Perhaps it can be transposed to the community leaders in that area, as a lot of people trust their RT (neighborhood unit) head, the priest, the religious leaders. The system as Indonesia is very unique.” This phenomenon is particularly pervasive in underdeveloped areas, where hierarchical interpersonal communication is still dominant.

### Mass media

When talking about mediated information, participants reported knowing about energy through various conventional and new media channels, ranging across nations, and age groups. News media was frequently mentioned to provide timely information via newspapers, TV news, and mobile news apps. Notably, the prevalence of cyber technology has altered people’s news attention to online portals, according to I2P7, who described news websites as “a breakfast friend, a lunch friend, a friend when relaxing.”

Some participants also mentioned various knowledge-intensive media sources, from textbooks and scientific publications (e.g., journals and magazines), to digital encyclopedias (e.g., Wikipedia). Some also talked about learning about energy by watching serial documentaries, especially the National Geographic (S2P9, M1P1, I3P1). Compared with text-based information that is cognitively demanding, the visualization of science through documentaries is more accessible and appealing to the average audience [90].

Entertainment media such as movies and TV programs also contain energy-related information. This kind of exposure to science is incidental, and the embedded information can either be intended or not. For instance, M2P2 came across an energy-related topic in the film Mission Impossible: “Even though it is an action movie but err, if we look at in between the lines, it teaches us how to use erm, air to generate energy, generate electricity.”

Lastly, participants showed heavy reliance on the Internet in acquiring energy information. Participants reported gaining information online mainly through search engines and social media platforms including Facebook, Instagram, Twitter, and YouTube.

Two reasons account for the informational reliance on social media rests in its networking nature. First, social media can spotlight trending topics, for instance, when a new technological invention comes out, like S1P2 said, “If there’s something going on, then I would see on like the trending page or something.” Second, influencers online can faithfully improve their followers’ awareness and interests in frontier science, triggering more information-seeking behaviors. I2P9 explained the endorsement and role model effect of influencers with her experience:

“I am a follower of Mr. Mardigu Wowiek (An Indonesian entrepreneur in oil and gas businesses.) Perhaps all of you know him. Mr. Mardigu is one of my role models. He seems to understand very much about nickel, about the earth’s sources, and he explained, pictured on Instagram about the condition of the earth. After this, I browsed what is this, what is this, nah, this helps a lot.”

Another point to address here is also motivation for acquiring information. As it was initially designed, search engines like Google used to require an actual search to find information. Now, empowered by big data and machine learning, search engines sometimes bring accurate recommendations for search or random popups, thus turning the role of the audience into passive receivers of information. This trend is even strengthened on social media platforms, as S1P10 noted, “Social media still has that effect of pushing to you what you often read, so I think that’s how I also get some additional articles.” Social media platforms provide spaces for users to both search and encounter information, like S1P10 explained, “When I use Facebook, I’m a little bit passive. Facebook brings it to me, but when I use YouTube, I go and seek it out.” This shows that users treat and experience various social media platforms in different ways.

### Overlaps and gaps in the information flows

RQ3 compares the information sources of the public with the information outlets of energy experts. In general, we found both overlaps and gaps and interestingly noted that some information agents are not synchronized or equally valued by the two groups.

### Overlaps

Scientists disseminate information to the public through several agents including education, mass media, and social media. Especially with education, energy information was well-mediated by schools, universities, and professional training sessions. However, we found that mass media—especially social media—have not been effectively utilized by energy experts. Mass media were availed by the scientists only on limited occasions, such as news interviews, while they were among the most sought-after channels for the public to know about energy, ranging from newspapers to TV documentaries. This mismatch shows the potential of media agents in enhancing science communication. For example, only one energy expert (I4P4) mentioned his use of podcasts and YouTube, suggesting the potential of online spaces for scientists to interact with the public directly.

### Gaps

Regarding the channels used, scientists mentioned their direct contact with the public via on-ground projects and road tour campaigns. The public, however, showed no experience or knowledge of such projects. Additionally, the public mentioned learning about energy through their personal experience, but this source of information was not reflected in the responses of energy experts.

Regarding connections with third-party stakeholders, scientists underlined their relationship with the government and other companies, whereas these stakeholders were absent from the public’s discussion. The public did not see these actors as sources for them to gain information. Moreover, they expressed concerns about the credibility of third parties, like the striking comment pointed at the government actors by S2P2, “The government will always have their own agenda, but scientists can maintain their scientific neutrality.” Others’ skepticism in the government rose from its shortness of expertise, as M1P3 said, “I do not really believe the ministry, because they are not in that field.”

A strong tie between the government and researchers can enhance public trust in the former. Those who see the government as endorsed by or working with research bodies tend to trust information from the government or energy-related departments (e.g., NEA, EMA, Singapore; KeTSA, Malaysia; ESDM, Indonesia). Like M1P1 said, “They [government] will not simply place people [researchers] who do not qualify.” A key finding is that governmental actors take their credibility largely from researchers working inside or behind; consequently, it is crucial to involve energy scientists in the communication process.

Nevertheless, the data revealed that the government-scientists alliance is far from perfect in providing information. Sometimes, scientists informed the government via documents or forums, giving them the responsibility of public communication. In Singapore, some scientists spoke of their experience of briefing and teaching the policymakers, like S4P7 said, “It has to be the government; it has to be the politicians who step forward and demonstrate that.” However, this is probably the view of scientists alone, as it contradicts the public’s belief that scientists are more reliable than the government in science communication. This displacement of duty signals the scientists to be more aware of their credibility and responsibility in disseminating knowledge, either independently or in cooperation with a third party.

## Discussion

This study mapped the gap between the public and the energy scientists by analyzing the channels for acquiring and disseminating energy-related information. Regarding the debate on the models used in science communication, this study examined the public-scientist engagement when it comes to energy-related information. Identifying information agents allowed us to explore their potential in facilitating science communication. Towards this end, we found more evidence for an indirect flow than a direct flow and that some agents are not fully utilized in disseminating energy-related information.

In the direct flow, scientists attempted to reach out to the public directly, and the communication is potentially two-way. However, for on-ground projects and campaigns to engage with the public, some scientists may be disappointed by the outcome of their efforts. The on-ground spread of information has always been a task in development communication [91]. In our study, the public’s scarce mention of direct contact with scientists showed the difficulty of direct communication. As a result, scientists should take better advantage of interactive media such as social media.

In the indirect flow, one-way communication was largely retained. Schooling as a socialization agent has received adequate attention from both scientists and the public. Nevertheless, information in this channel moves relatively slowly and cannot continue after one’s school term; recent developments may also take time to be incorporated in formal modules and courses. To keep the public constantly informed, more enduring and efficient channels have been brought forward, such as the media.

Digital technologies have removed barriers of space and time in science communication [92]. In our study, the public participants reported getting information from the Internet (e.g., news websites, social media). The public showed their demand for and trust in information sourced directly from scientists, which shows the active role of the public in information exchange, as well as the deeper involvement of the scientists in providing original information.

Despite the current social media use of some scientists, their primary goals have been to boost interdisciplinary collaborations [93], to build online communities of interest groups [94], or to gather more information [95]. Social media use for public communication of science is underutilized. To improve their direct relationship with the public, scientists should leverage on the power of multiple online platforms and develop purposeful strategies to reach diverse populations. The public tends to seek professionalism, expertise, and neutrality, which scientists and experts can provide. However, this may also be a matter of resources and training. First, unlike government offices, scientists may not have the time, manpower, or network to routinely access media organizations. Second, they may also lack training in public communication and dealing with journalists. Thus, the active role of mass media and journalism can come to the forefront through a direct collaboration with the scientists.

Additionally, scientists should also put forth their opinions by strengthening their alliance with third parties. First, they can work with the government, which usually enjoys more coverage in the mainstream media. Our results show that the presence or absence of scientists in energy policy reports from the government may influence people’s confidence in the policy. Therefore, government by itself may not serve as a credible source of information, and should include scientists’ expert opinions in their media statements or public communication. Similarly, while corporate sectors have invested a lot in developing energy projects, the public tends to be suspicious about information from them. Scientists, often perceived by the public as independent, may be more credible sources of information even for business-related energy claims, something that future studies can continue to probe.

Several implications are made for future research, given the limitations of the current study. The first limitation to address is the remainder of the current map of information sources. Although our recruitment adhered to the cluster sampling for both the public and the expert participants, we still could not achieve the maximum variation of the population. We acknowledge the interview-based method cannot exhaust all the relevant channels, leaving gaps for future research to fill, such as by conducting nationwide surveys. Indeed, the results presented here, while grounded in qualitative data, cannot be generalized to the population at large. While generalization is not the goal of this qualitative inquiry, future studies can build on the results presented here to assess trends in the population using national surveys. Second, it is also necessary to specify the difficulties scientists face in communicating knowledge and their motivation for undertaking this task. We found some experts do not see themselves responsible for and capable of communicating science, while others are actively exercising such skills. What brings the different motivations? How does motivation affect scientists’ performance in public communication? These queries should be answered in more focused studies on experts across fields. The last implication points at the role of other science communicators. Another paper from our project examined environmental activists’ role in energy communication. However, it is still unclear how these external actors can boost the connection between scientists and the public and how the public perceives their credibility. As we suggested, the interplays of scientists, the public, and third-party communicators require further insights. Through these, future studies can look into establishing an improved dissemination and reception system of science information to make the public engaged with alternative and emerging energies, including those that have been around but remain underutilized, such as biomass energy.
